# Expression of Caspases in the Pig Endometrium Throughout the Estrous Cycle and at the Maternal-Conceptus Interface During Pregnancy and Regulation by Steroid Hormones and Cytokines

**DOI:** 10.3389/fvets.2021.641916

**Published:** 2021-02-12

**Authors:** Wonchul Jung, Inkyu Yoo, Jisoo Han, Minjeong Kim, Soohyung Lee, Yugeong Cheon, Minsun Hong, Bo-Young Jeon, Hakhyun Ka

**Affiliations:** ^1^Department of Biological Science and Technology, Yonsei University, Wonju, South Korea; ^2^Department of Biomedical Laboratory Science, Yonsei University, Wonju, South Korea

**Keywords:** pig, endometrium, apoptosis, caspase, differentiation

## Abstract

Caspases, a family of cysteine protease enzymes, are a critical component of apoptotic cell death, but they are also involved in cellular differentiation. The expression of caspases during apoptotic processes in reproductive tissues has been shown in some species; however, the expression and regulation of caspases in the endometrium and placental tissues of pigs has not been fully understood. Therefore, we determined the expression of caspases *CASP3, CASP6, CASP7, CASP8, CASP9*, and *CASP10* in the endometrium throughout the estrous cycle and pregnancy. During the estrous cycle, the expression of all caspases and during pregnancy, the expression of *CASP3, CASP6*, and *CASP7* in the endometrium changed in a stage-specific manner. Conceptus and chorioallantoic tissues also expressed caspases during pregnancy. CASP3, cleaved-CASP3, and CASP7 proteins were localized to endometrial cells, with increased levels in luminal and glandular epithelial cells during early pregnancy, whereas apoptotic cells in the endometrium were limited to some scattered stromal cells with increased numbers on Day 15 of pregnancy. In endometrial explant cultures, the expression of some caspases was affected by steroid hormones (estradiol-17β and/or progesterone), and the cytokines interleukin-1β and interferon-γ induced the expression of *CASP3* and *CASP7*, respectively. These results indicate that caspases are dynamically expressed in the endometrium throughout the estrous cycle and at the maternal-conceptus interface during pregnancy in response to steroid hormones and conceptus signals. Thus, caspase action could be important in regulating endometrial and placental function and epithelial cell function during the implantation period in pigs.

## Introduction

The structure and function of the uterus changes significantly during the reproductive cycle and pregnancy in mammalian species. The degree of change in the endometrium during the cycle varies by species, with the most dramatic changes found in humans and non-human primates, which form a hemochorial type placenta (1–3). In pigs, which form a true epitheliochorial type placenta, the endometrium also undergoes morphological and functional change during the estrous cycle and pregnancy (4). During the estrous cycle in pigs, endometrial change is affected mainly by the ovarian steroid hormones estrogen and progesterone (5, 6), and during early pregnancy, it is driven by conceptus-derived signals, including estrogen and the cytokines interleukin-1β (IL1B), interferon-δ (IFND), and interferon-γ (IFNG), in addition to ovarian steroid hormones (4, 7, 8).

Apoptosis, a programmed cell death, plays a critical role in a variety of physiological processes in multicellular organisms. For example, it maintains functional tissue homeostasis by eliminating unwanted or dysfunctional cells (9, 10). Apoptosis occurs in the endometrium during the estrous cycle and pregnancy to regulate endometrial homeostasis (11, 12). In the human endometrium, apoptotic cell death is observed in endometrial epithelial and stromal cells, with a higher apoptotic rate in the late secretory to early proliferative phases than in the late proliferative to mid-secretory phases of the menstrual cycle (13). In pigs, cells undergoing apoptosis are detected mainly in endometrial stroma during the estrous cycle and early pregnancy and in luminal epithelial cells at the proestrus phase of the estrous cycle, but apoptotic cell death does not occur as dramatically in pigs as it does in primates during the reproductive cycle (14).

Apoptotic cell death is induced by intrinsic and extrinsic pathways. The intrinsic pathway is mediated by various intracellular stress and mitochondrial factors, whereas the extrinsic pathway is triggered by extracellular death signals, such as tumor necrosis factor (TNF) superfamily members: TNF-α, Fas ligand (FASLG), and TNF-related apoptosis-inducing ligand (TRAIL, also known as TNFSF10) (15, 16). The two pathways result in the activation of caspases, which are cytoplasmic cysteine protease enzymes, to induce apoptotic cell death. Caspases play essential roles in apoptosis and inflammation and are divided into two groups, initiator caspases (CASP8, CASP9, and CASP10) and executioner caspases (CASP3, CASP6, and CASP7) (9, 17, 18). Once the executioner caspases are activated by the initiator caspases, they recognize the aspartic residue of various intracellular target proteins and cleave them to cause apoptotic cell death. In that way, caspases are used as a representative marker for cells in which apoptosis has occurred. However, the apoptotic signaling pathway that activates caspases also plays an important role in the differentiation of various cell types, such as immune cells, trophoblasts, spermatocytes, epithelial cells, and stem cells (19, 20). It has been suggested that caspase activation is locally regulated during cellular remodeling without causing apoptotic cell death and that transient caspase activity is used for cell fate determination (10, 21).

Although endometrial changes during the estrous cycle and pregnancy involve the apoptotic process and the function of caspases is essential during apoptotic cell death and cellular differentiation, the pattern of caspase expression in the endometrium during the estrous cycle and pregnancy is not fully understood in pigs. We hypothesized that caspases are expressed in the endometrium during the estrous cycle and at the maternal-conceptus interface during pregnancy to regulate apoptosis and cellular differentiation. Therefore, we determined in pigs (1) the expression of caspases (*CASP3, CASP6, CASP7, CASP8, CASP9*, and *CASP10*) in the endometrium during the estrous cycle and pregnancy, conceptus tissues during early pregnancy, and chorioallantoic tissues during mid- to late pregnancy; (2) the localization of caspases and apoptotic cells in the endometrium; and (3) the regulation of caspase expression by the steroid hormones estrogen and progesterone and by the cytokines IL1B and IFNG in endometrial tissues.

## Materials and Methods

### Animals and Tissue Preparation

All experimental procedures involving animals were conducted in accordance with the Guide for the Care and Use of Research Animals in Teaching and Research and approved by the Institutional Animal Care and Use Committee of Yonsei University and the National Institute of Animal Science. Sexually mature Landrace and Yorkshire crossbred female gilts of similar age (6–8 months) and weight (100–120 kg) were assigned randomly to either cyclic or pregnant status, as described previously (22). Gilts assigned to the pregnant uterus status group were artificially inseminated with fresh boar semen at the onset of estrus (Day 0) and 12 h later. The reproductive tracts of the gilts were obtained immediately after slaughter on Days 0, 3, 6, 9, 12, 15, or 18 of the estrous cycle or Days 10, 12, 15, 30, 60, 90, or 114 of pregnancy (*n* = 3–6/day/status). Pregnancy was confirmed by the presence of apparently normal filamentous conceptuses in uterine flushings on Days 10, 12, and 15 and the presence of embryos and placenta on later days of pregnancy. Conceptus tissues were obtained from uterine flushings on Days 12 and 15 of pregnancy. Uterine flushings were obtained by introducing and recovering 25 ml of phosphate-buffered saline (PBS; pH 7.4) into each uterine horn. Chorioallantoic tissues were obtained on Days 30, 60, 90, and 114 of pregnancy (*n* = 3–4/day). Endometrial tissues from prepubertal gilts (*n* = 8; approximately 6 months of age) that had not undergone the estrous cycle, with no corpus luteum formed, were obtained from a local slaughterhouse. Endometrium, dissected free of myometrium, was collected from the middle portion of each uterine horn, snap-frozen in liquid nitrogen, and stored at −80°C prior to RNA extraction. For immunohistochemistry, cross-sections of the endometrium were fixed in 4% paraformaldehyde in PBS (pH 7.4) for 24 h and then embedded in paraffin as previously described (23).

### Explant Cultures

To determine the effects of steroid hormones, IL1B, and IFNG on the expression of caspase mRNA in the endometrium, endometrial tissue was dissected from the myometrium and placed into warm phenol red-free Dulbecco's modified Eagle's medium/F-12 (DMEM/F-12) (Sigma) containing penicillin G (100 IU/ml) and streptomycin (0.1 mg/ml), as described previously (23–25) with some modifications. The endometrium was minced with scalpel blades into small pieces (2–3 mm^3^), and 500 mg were placed into T25 flasks with serum-free modified DMEM/F-12 containing 10 μg/ml insulin (Sigma), 10 ng/ml transferrin (Sigma), and 10 ng/ml hydrocortisone (Sigma). To analyze the effect of steroid hormones on the expression of caspases, endometrial explants from immature gilts, immediately after mincing, were cultured with rocking in the presence of increasing doses of estradiol-17β (E_2_; 0, 5, 50, or 500 pg/ml; Sigma) or progesterone (P_4_; 0, 0.3, 3, or 30 ng/ml; Sigma) for 24 h in an atmosphere of 5% CO_2_ in air at 37°C. The doses were chosen to encompass the full concentration range of physiological levels of E_2_ and P_4_ in the endometrium during the estrous cycle and pregnancy (8). To analyze the effect of IL1B on *CASP3* and the effect of IFNG on *CASP7* expression, endometrial explant tissues from Day 12 of the estrous cycle were treated with E_2_ (10 ng/ml), P_4_ (30 ng/ml), and increasing doses of IL1B (0, 1, 10, and 100 ng/ml; Sigma) or IFNG (0, 1, 10, and 100 ng/ml; R&D Systems, Minneapolis, MN, USA) at 37°C for 24 h. To determine the effect of the steroid hormones on the expression of *CASP3* during the implantation period, endometrial explant tissues from Day 12 of the estrous cycle were treated with ethanol (control), E_2_ (10 ng/ml; Sigma, USA), P_4_ (30 ng/ml; Sigma, USA), P_4_+E_2_, P_4_+E_2_+ICI182,780 (ICI; an estrogen receptor antagonist; 200 ng/ml; Tocris Bioscience, Ellisville, MO, USA), or P_4_+E_2_+RU486 (RU; a progesterone receptor antagonist; 30 ng/ml; Sigma, USA) for 24 h. The explant tissues were then harvested, and total RNA was extracted for a real-time RT-PCR analysis to determine the expression levels of caspase mRNA. These experiments were conducted using endometrium from three gilts on Day 12 of the estrous cycle in triplicate and eight immature gilts.

### Total RNA Extraction, Reverse Transcription-Polymerase Chain Reaction (RT-PCR), and Cloning of Porcine Caspase cDNA

Total RNA was extracted from endometrial and conceptus tissues using TRIzol reagent (Invitrogen, Carlsbad, CA, USA) according to the manufacturer's recommendations, as described previously (22). The quantity of RNA was assessed spectrophotometrically, and RNA integrity was validated following electrophoresis in 1% agarose gel. Four micrograms of total RNA from endometrial, conceptus, and chorioallantoic tissues were treated with DNase I (Promega, Madison, WI, USA) and reverse transcribed using SuperScript II Reverse Transcriptase (Invitrogen) to obtain cDNA. The cDNA templates were then diluted at a 1:4 ratio with sterile water and amplified by PCR using Taq polymerase (Takara Bio, Shiga, Japan) and specific primers based on porcine caspase mRNA sequences. The PCR conditions, sequences of primer pairs for caspases, and expected product sizes are listed in Supplementary Table 1. The PCR products were separated on 2% agarose gel and visualized by ethidium bromide staining. The identity of each amplified PCR product was verified by sequence analysis after cloning into the pCRII vector (Invitrogen).

### Quantitative Real-Time RT-PCR

To analyze the levels of caspase expression in the endometrial and chorioallantoic tissues, real-time RT-PCR was performed using an Applied Biosystems StepOnePlus System (Applied Biosystems, Foster City, CA, USA) with the SYBR Green method, as described previously (22). Complementary DNA was synthesized from 4 μg of total RNA isolated from different uterine endometrial and chorioallantoic tissues, and the newly synthesized cDNA (total volume of 21 μl) was diluted 1:4 with sterile water and used for PCR. Power SYBR Green PCR Master Mix (Applied Biosystems) was used for the PCR reactions. The final reaction volume of 20 μl contained 2 μl of cDNA, 10 μl of 2× Master mix, 2 μl of each primer, and 4 μl of distilled H_2_O. The annealing temperature and number of cycles for PCR were the same for all products obtained. The results are reported as expression relative to that detected on Day 0 of the estrous cycle, that on Day 30 of pregnancy in chorioallantoic tissues, or that in control explant tissues after normalization of the transcript amount to the geometric mean of endogenous porcine ribosomal protein L7 (*RPL7*) and ubiquitin B (*UBB*), and TATA binding protein (*TBP*) controls, all using the 2^−ΔΔCT^ method as previously described (26).

### Immunohistochemical Analysis

To identify the type(s) of porcine endometrial cells expressing CASP3, cleaved-CASP3, CASP7, poly (ADP-ribose) polymerase (PARP1), an enzyme that is cleaved during apoptosis and used as a hallmark for apoptosis (27), and cleaved-PARP1, sections were immunostained. Sections (5 μm thick) were deparaffinized and rehydrated in an alcohol gradient. Tissue sections were boiled in citrate buffer (pH 6.0) for 10 min. Then, they were washed with PBST (PBS with 0.1% Tween-20) three times, and a peroxidase block was performed with 0.5% (v/v) H_2_O_2_ in methanol for 30 min. Tissue sections were then blocked with 10% normal goat serum for 30 min at room temperature. Rabbit polyclonal anti-CASP3 antibody (5 μg/ml; Cell Signaling, Danvers, MA, USA), rabbit polyclonal anti-cleaved-CASP3 antibody (5 μg/ml; Cell Signaling), mouse monoclonal anti-CASP7 antibody (5 μg/ml; Enzo Life Sciences, Farmingdale, NY, USA), rabbit polyclonal anti-PARP1 antibody (1 μg/ml; Santa Cruz Biotechnology, Santa Cruz, CA, USA), or rabbit monoclonal anti-cleaved-PARP1 antibody (1 μg/ml; GeneTex, Irvine, CA, USA) were added and incubated overnight at 4°C in a humidified chamber. For each tissue tested, purified normal rabbit IgG or mouse IgG was substituted for the primary antibody as a negative control. Tissue sections were washed with PBST three times. Biotinylated goat anti-rabbit or anti-mouse secondary antibody (1 μg/ml; Vector Laboratories, Burlingame, CA, USA) was added and incubated for 1 h at room temperature. Following washes with PBST, a streptavidin peroxidase conjugate (Invitrogen) was added to the tissue sections, which were then incubated for 10 min at room temperature. The sections were washed with PBST, and aminoethyl carbazole substrate (Invitrogen) was added to the tissue sections, which were then incubated for 10 min at room temperature. The tissue sections were washed in water, counterstained with Mayer's hematoxylin, and coverslipped. Images were captured using an Eclipse TE2000-U microscope (Nikon, Seoul, Korea) and processed with Adobe Photoshop CS6 software (Adobe Systems, Seattle, WA, USA).

### TUNEL Assay and Immunofluorescence

Apoptotic cells in endometrial tissue sections were analyzed using the terminal deoxynucleotidyl transferase-mediated dUTP nick end labeling (TUNEL) assay with an In Situ Cell Death Detection Kit (Roche Diagnostics, Mannheim, Germany) used according to the manufacturer's recommendations, as described previously (28). Endometrial tissue sections (5 μm thick) were deparaffinized and rehydrated in an alcohol gradient. The sections were then boiled with 0.1 M citrate buffer (pH 6.0) for 3 min, cooled at room temperature for 10 min, and then washed three times in PBS. For a positive control for TUNEL staining, the sections were treated with DNase I (3 U/ml; Promega) in 50 mM Tris-HCl (pH 7.5), 10 mM MgCl_2_, and 1 mg/ml bovine serum albumin (BSA; Bovogen Biologicals, Melbourne, Australia) for 10 min at room temperature and then washed with PBS. Tissue sections were then blocked with 0.1 M Tris-HCl (pH 7.5) containing 3% (w/v) BSA and 20% (v/v) normal bovine serum for 30 min at room temperature. The TUNEL reaction was performed according to the kit instructions. After the TUNEL reactions, tissue sections were washed with PBS. The tissue sections were counterstained with 4',6-diamidino-2-phenylindole (DAPI), and fluorescence images were captured using an Eclipse TE2000-U microscope (Nikon, Seoul, Korea) with Adobe Photoshop CS6 software (Adobe Systems, Seattle, WA, USA).

### Statistical Analysis

Data from real-time RT-PCR for caspase expression were subjected to ANOVA using the general linear models procedures in SAS (Cary, NC, USA). As sources of variation, the model included day, pregnancy status (cyclic or pregnant, Days 12 and 15 post-estrus), and their interactions to evaluate steady-state levels of caspase mRNA. Data from real-time RT-PCR performed to assess the effects of day of the estrous cycle (Days 0, 3, 6, 9, 12, 15, and 18) and pregnancy (Days 10, 12, 15, 30, 60, 90, and 114) and the effects of day of pregnancy (Days 30, 60, 90, and 114) on chorioallantoic tissues were analyzed using a least squares regression analysis. The effects of E_2_, P_4_, IL1B, and IFNG doses on explant cultures were analyzed by one-way ANOVA followed by Tukey's post-test. Data from real-time RT-PCR to assess the effects of steroid hormones and their receptor antagonists on explant culture were analyzed by preplanned orthogonal contrasts (control vs. E_2_; control vs. P_4_; P_4_ vs. P_4_+E_2_; P_4_+E_2_ vs. P_4_+E_2_+ICI; and P_4_+E_2_ vs. P_4_+E_2_+RU). Data are presented as means with standard error of the mean. A *P*-value <0.05 was considered significant, and *P*-values 0.05–0.10 were considered to indicate a trend toward significance.

## Results

### Expression of Caspase mRNA in the Endometrium During the Estrous Cycle and Pregnancy

**Figure 1:**
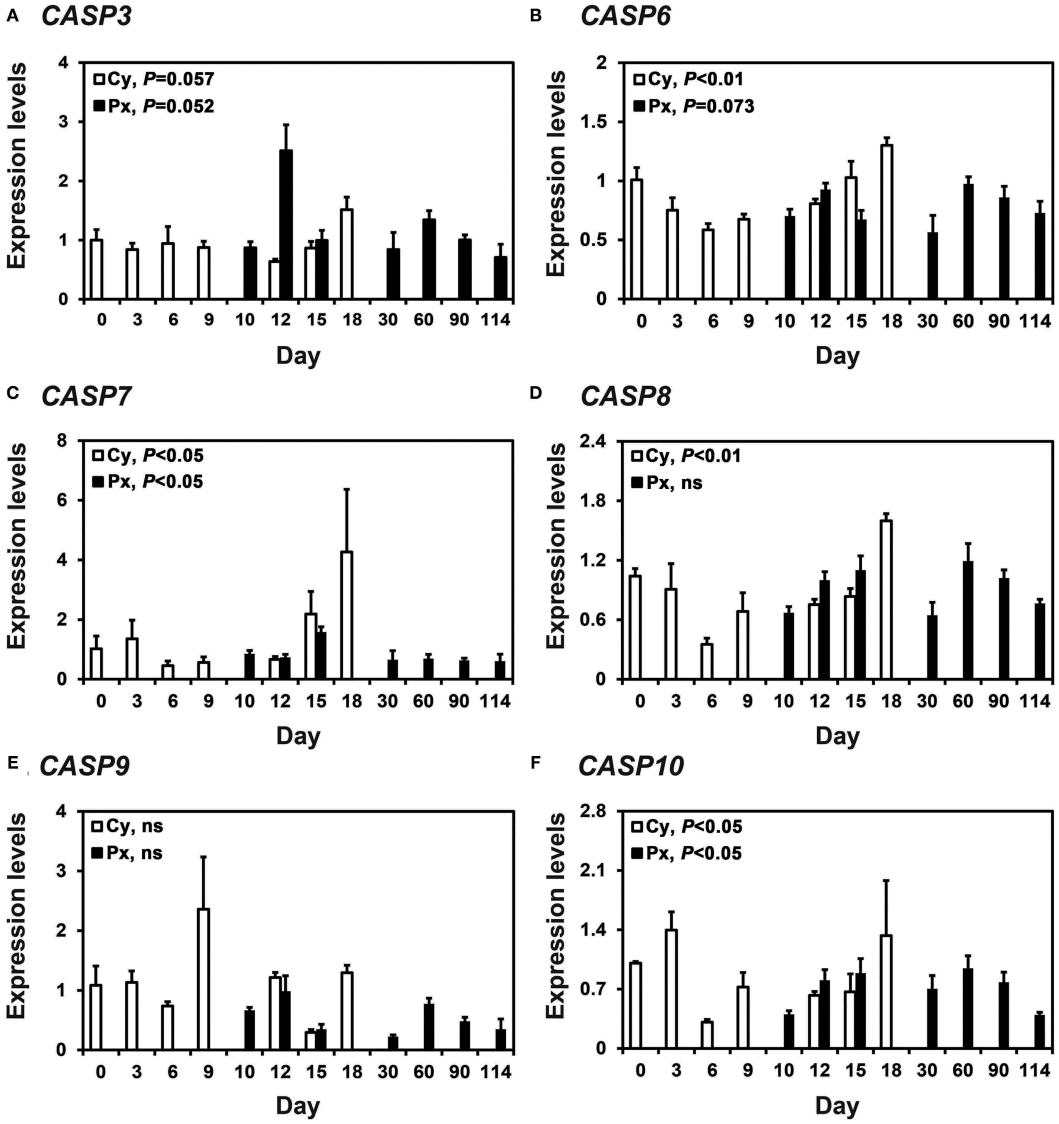
Expression of *CASP3* **(A)**, *CASP6* **(B)**, *CASP7* **(C)**, *CASP8* **(D)**, *CASP9* **(E)**, and *CASP10* **(F)** mRNA in the endometrium during the estrous cycle and pregnancy in pigs. Endometrial tissue samples from cyclic (Cy) and pregnant (Px) gilts were analyzed by real-time RT-PCR, and data are reported as the expression relative to that detected on Day 0 of the estrous cycle after normalization to the transcript amount of the endogenous *RPL7, UBB*, and *TBP* mRNAs. Data are presented as the mean with standard error. Statistical significances for the effect of day during the estrous cycle and pregnancy are indicated; ns, not significant.

In real-time RT-PCR analyses, we found that *CASP3, CASP6, CASP7, CASP8, CASP9*, and *CASP10* mRNA was expressed in the endometrium during the estrous cycle and pregnancy (Figure 1). During the estrous cycle, the steady-state levels of *CASP3* (quadratic, *P* = 0.0572), *CASP6* (quadratic, *P* < 0.01), *CASP7* (linear, *P* < 0.05), *CASP8* (quadratic, *P* < 0.01), and *CASP10* (quadratic, *P* < 0.05) mRNA changed, with the highest levels of *CASP3, CASP6, CASP7*, and *CASP8* in the proestrus phase and that of *CASP10* in the proestrus to metestrus phase. On Days 12 and 15 post-estrus, the expression of *CASP3* was affected by day (*P* < 0.05), status (*P* < 0.01), and the day x status interaction (*P* < 0.05). The expression of *CASP6* was affected by the day x status interaction (*P* < 0.05), that of *CASP7* was affected by day (*P* < 0.05), that of *CASP8* was affected by status (*P* < 0.01), and that of *CASP9* was affected by day (*P* < 0.01). The expression of *CASP10* was not affected by day, status, or the day x status interaction. During pregnancy, the steady-state levels of *CASP3* (linear, *P* = 0.0526), *CASP6* (cubic, *P* = 0.073), *CASP7* (linear, *P* < 0.05), and *CASP10* (quadratic, *P* < 0.05), but not *CASP8* or *CASP9* mRNA, changed with the highest levels on Day 12 for *CASP3*, on Day 15 for *CASP7*, and Day 60 for *CASP6* and *CASP10*.

### Expression of Caspase mRNA in Conceptuses During Early Pregnancy and Chorioallantoic Tissues in Later Stages of Pregnancy

**Figure 2:**
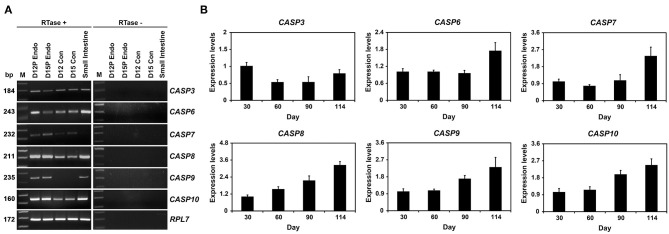
Expression of *CASP3, CASP6, CASP7, CASP8, CASP9*, and *CASP10* by conceptuses from Days 12 and 15 of pregnancy **(A)** and chorioallantoic tissues during mid- to late pregnancy **(B)**. **(A)** RT-PCR analyses of *CASP3, CASP6, CASP7, CASP8, CASP9*, and *CASP10* mRNA in conceptuses on Days 12 and 15 of pregnancy were performed using total RNA preparations. *RPL7* was used as a positive control. RTase ±, with (+) or without (–) reverse transcriptase; M, molecular marker; D12 Endo, endometrium on day 12 of pregnancy; D12 Con, Day 12 conceptus; D15 Con, Day 15 conceptus. **(B)**. Real-time RT-PCR analyses of the expression of *CASP3, CASP6, CASP7, CASP8, CASP9*, and *CASP10* mRNA in chorioallantoic tissues on Days 30, 60, 90, and 114 of pregnancy. Data are reported as expression relative to that detected on Day 30 of pregnancy after normalization to the transcript amount of the endogenous *RPL7, UBB*, and *TBP* mRNAs control, and data are presented as means with standard errors.

In RT-PCR analysis using cDNAs from conceptuses from Days 12 and 15 of pregnancy, we found that *CASP3, CASP6, CASP7, CASP8*, and *CASP10* mRNA but not *CASP9* mRNA in conceptuses from both days of early pregnancy (Figure 2A). These caspases were also detectable in endometrial tissues from same days. In addition, we performed real-time RT-PCR analyses to determine whether the expression of *CASP3, CASP6, CASP7, CASP8, CASP9*, and *CASP10* mRNA changed in chorioallantoic tissues during pregnancy. The abundance of *CASP3, CASP6, CASP7, CASP8, CASP9*, and *CASP10* mRNA in chorioallantoic tissues changed, with the highest levels on Day 30 for *CASP3* and at term for *CASP6, CASP7, CASP8, CASP9*, and *CASP10* (linear effect of day for *CASP6, CASP7, CASP8, CASP9*, and *CASP10, P* < 0.01; quadratic effect of day for *CASP3, P* < 0.01) (Figure 2B).

### Localization of CASP3, Cleaved-CASP3, and CASP7 Proteins in the Endometrium on Days 12 and 15 Post-estrus

**Figure 3:**
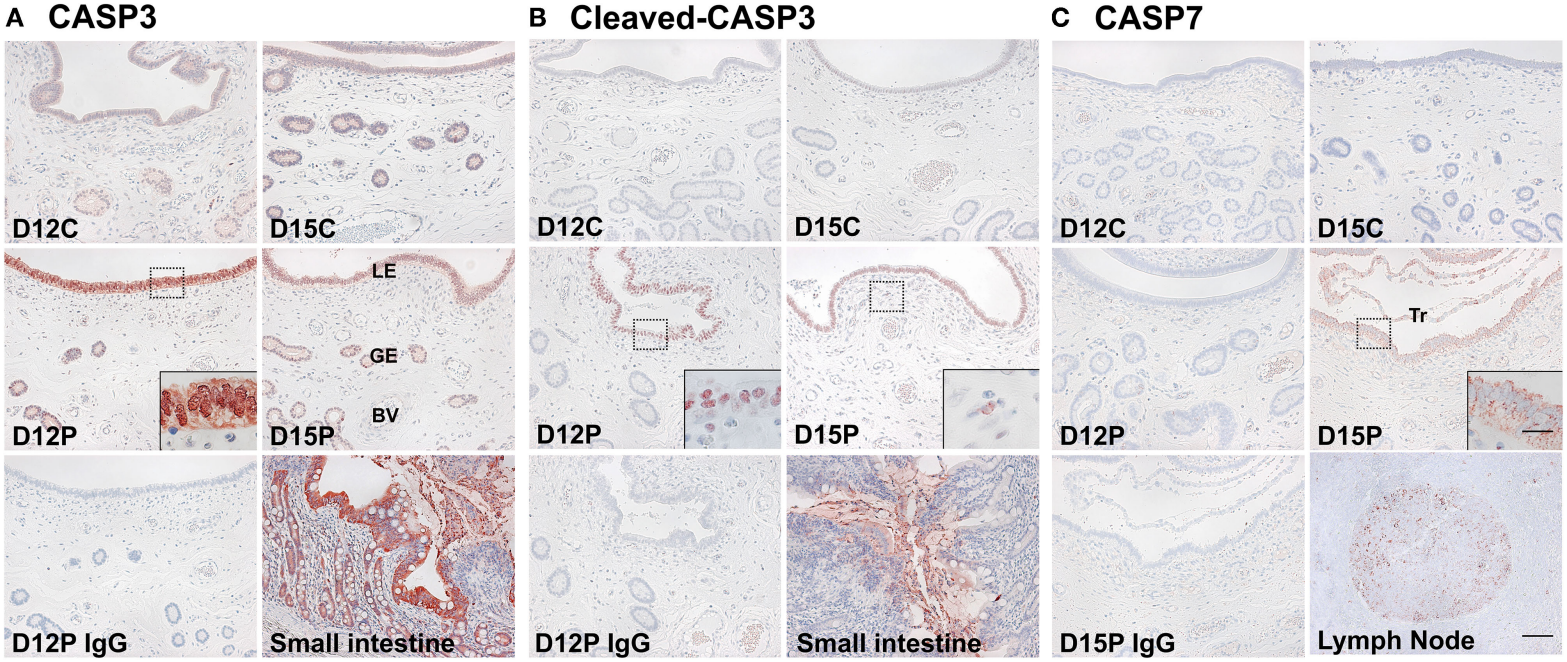
Immunohistochemical localization of CASP3 **(A)**, cleaved-CASP3 **(B)**, and CASP7 **(C)** proteins in the endometrium on Days 12 and 15 post-estrus. Representative uterine sections from Days 12 or 15 of pregnancy immunostained with normal IgG are shown as negative controls, and tissue sections from the small intestine and lymph node serve as positive controls for CASP3, cleaved-CASP3, and CASP7 immunostaining. D, Day; C, estrous cycle; P, pregnancy; LE, luminal epithelium; GE, glandular epithelium; BV, blood vessel; Tr, trophectoderm. Bars = 100 μm and 20 μm in insets.

Having determined that *CASP3, CASP6, CASP7, CASP8, CASP9*, and *CASP10* mRNA was present in the endometrium during the estrous cycle and pregnancy and in conceptuses and chorioallantoic tissues during pregnancy and that the expression of *CASP3* and *CASP7* mRNA was highest during early pregnancy, we next determined the cellular localization of the CASP3, cleaved-CASP3 (an active form), and CASP7 proteins in the endometrium on Days 12 and 15 post-estrus using immunohistochemistry (Figure 3). CASP3 proteins were mainly detected in endometrial luminal (LE) and glandular epithelial (GE) cells and in scattered stromal cells, with stronger signal intensity on Days 12 and 15 of pregnancy than during the estrous cycle, and they were localized subcellularly to both the cytoplasm and the nucleus (Figure 3A). The active form of CASP3, cleaved-CASP3 protein, was localized primarily to the nucleus of LE cells and some stromal cells in the endometrium on Days 12 and 15 of pregnancy (Figure 3B). Both CASP3 and cleaved-CASP3 proteins were detected in the small intestine used as a positive control. CASP7 protein was localized to the cytoplasm of LE and stromal cells in the endometrium, but only on Day 15 of pregnancy (Figure 3C). Trophectoderm cells in conceptuses were also positive for CASP7 protein on Day 15 of pregnancy (Figure 3C). CASP7 protein was detected in the lymph node used as a positive control. Immunohistochemistry for cleaved-CASP7 was not done due to the lack of an appropriate antibody to detect porcine cleaved-CASP7 protein.

### TUNEL Staining and PARP Cleavage Analysis for *in situ* Apoptotic Cell Death in the Endometrium During the Estrous Cycle and Pregnancy

**Figure 4:**
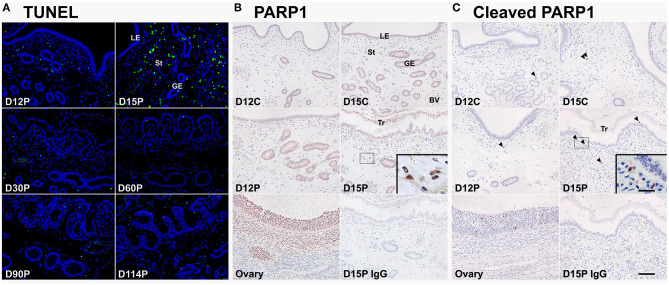
TUNEL staining **(A)** and immunohistochemical localization of PARP1 **(B)** and cleaved-PARP1 **(C)** proteins for *in situ* apoptotic cell death in the endometrium during the estrous cycle and pregnancy. Cells undergoing apoptosis in the endometrium during pregnancy were localized using the TUNEL assay (green), and tissue morphology is shown by DAPI staining. Representative uterine sections from Day 15 of pregnancy immunostained with normal IgG are shown as negative control and tissue sections from the ovary serve as positive controls for PARP1 and cleaved-PARP1 immunostaining. D, day; P, pregnancy; LE, luminal epithelium; GE, glandular epithelium; St, stroma; BV, blood vessel; Tr, trophectoderm. Arrowheads indicate cleaved-PARP1-positive cells. Bar = 100 μm and 20 μm in inset.

Because CASP3 and CASP7 proteins were localized to endometrial epithelial and stromal cells during the estrous cycle and pregnancy, we determined whether cells expressing CASP3 and CASP7 were undergoing apoptotic cell death. Because apoptotic cells undergo DNA degradation and PARP1, an enzyme involved in DNA repair, is cleaved by caspases (29), we performed the TUNEL assay and immunostaining of PARP1 and cleaved-PARP1 in endometrial tissues from pregnant pigs. We found that apoptotic cells in the endometrium during pregnancy were predominantly in stromal cells, not in epithelial cells, with many apoptotic cells found on Day 15 of pregnancy and very few cells found during the later stages of pregnancy (Figure 4A). PARP1 protein was localized to most cell types in the endometrium on Days 12 and 15 of the estrous cycle and pregnancy (Figure 4B), but cleaved-PARP1, a marker for apoptotic cells, was localized primarily to stromal cells on Day 15 of pregnancy (Figure 4C). The PARP1 and cleaved-PARP1 proteins were also detected in the ovary used as a positive control.

### Effects of the Steroid Hormones E_2_ and P_4_ on Caspase Expression in Endometrial Tissue of Prepubertal Gilts

**Figure 5:**
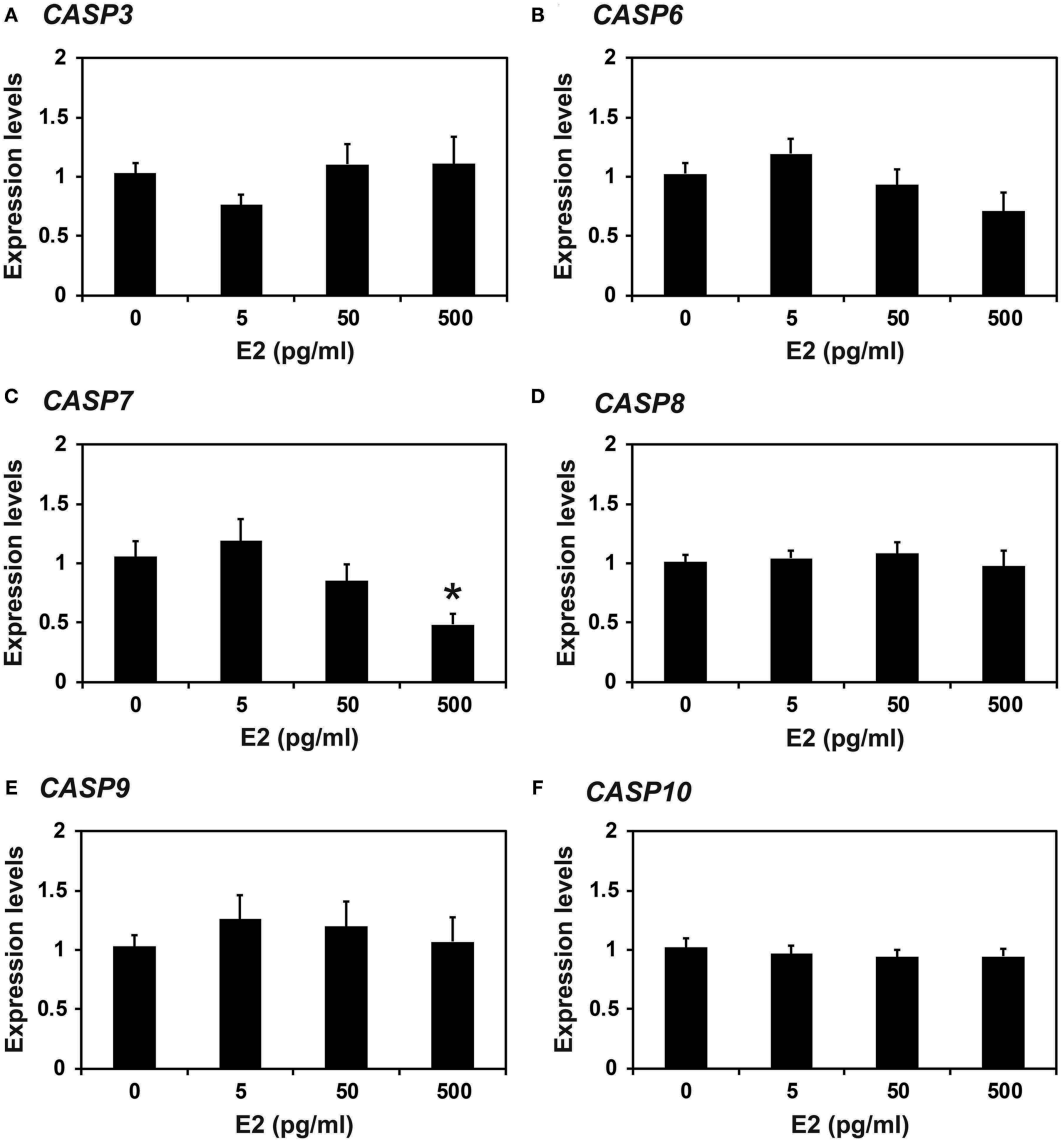
Effect of estradiol on *CASP3* **(A)**, *CASP6* **(B)**, *CASP7* **(C)**, *CASP8* **(D)**, *CASP9* **(E)**, and *CASP10* **(F)** mRNA in endometrial explant cultures. Endometrial explants from immature gilts were cultured at 37°C in DMEM/F-12 with increasing doses of estradiol-17β (E_2_; 0, 5, 50, and 500 pg/ml) for 24 h. Experiments were performed with endometria from eight gilts. The abundance of mRNA, determined by real-time RT-PCR, is relative to that of *CASP3, CASP6, CASP7, CASP8, CASP9*, and *CASP10* mRNA in the control group of endometrial explants (0 pg/ml E_2_) after normalization to the transcript amount of *RPL7, UBB*, and *TBP* mRNAs. Data are presented as the mean with standard error. The asterisk denotes statistically significant difference when values were compared with the control group: *P < 0.05.

**Figure 6:**
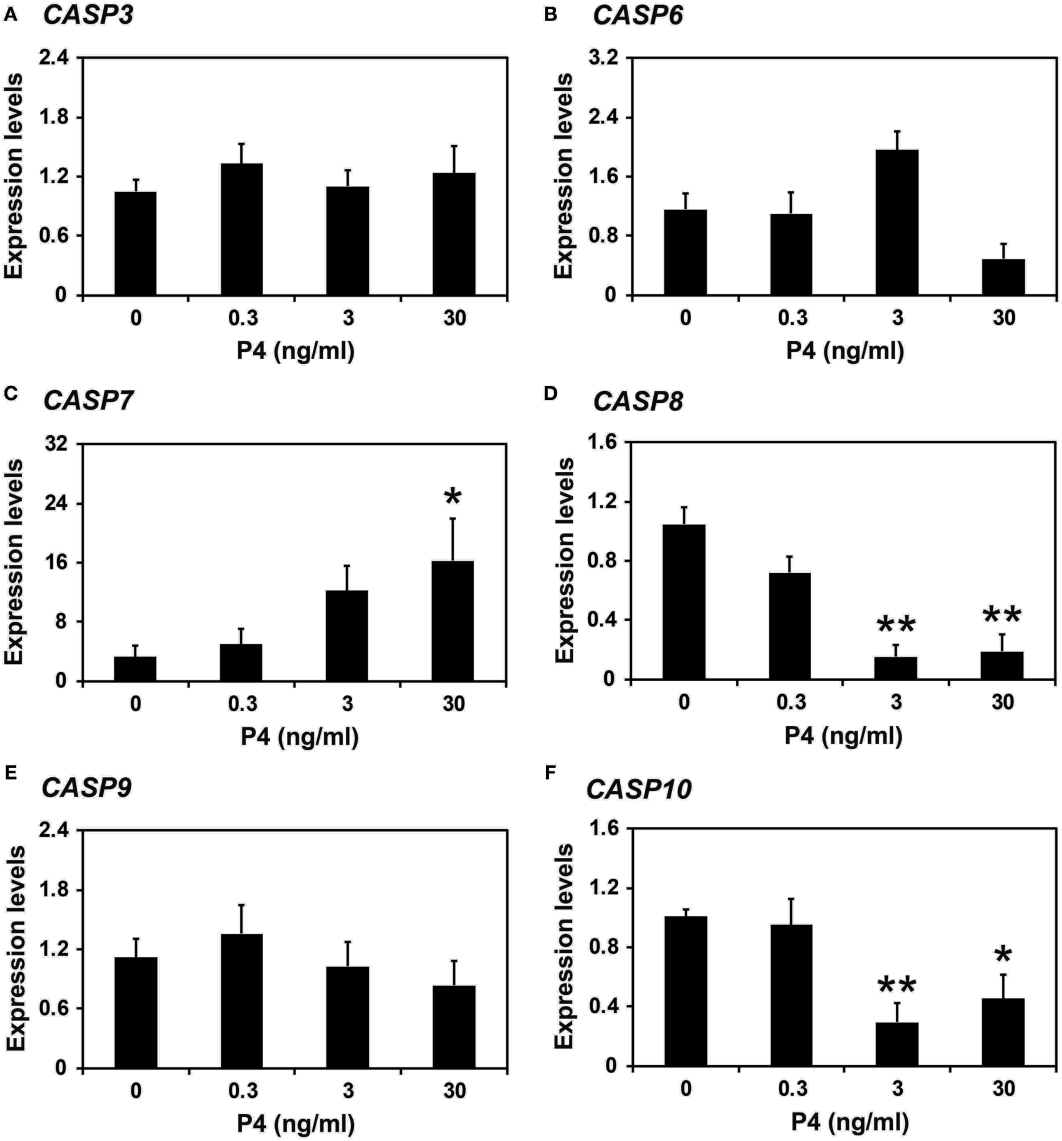
Effect of progesterone on *CASP3* **(A)**, *CASP6* **(B)**, *CASP7* **(C)**, *CASP8* **(D)**, *CASP9* **(E)**, and *CASP10* **(F)** mRNA in endometrial explant cultures. Endometrial explants from immature gilts were cultured at 37°C in DMEM/F-12 with increasing doses of progesterone (P_4_; 0, 0.3, 3, and 30 ng/ml) for 24 h. Experiments were performed with endometria from eight gilts. The abundance of mRNA, determined by real-time RT-PCR, is relative to that of *CASP3, CASP6, CASP7, CASP8, CASP9*, and *CASP10* mRNA in the control group of endometrial explants (0 ng/ml P_4_) after normalization to the transcript amount of *RPL7, UBB*, and *TBP* mRNAs. Data are presented as the mean with standard error. The asterisks denote statistically significant differences when values were compared with the control group: *P < 0.05; **P < 0.01.

Because the expression of caspases changed during the estrous cycle and because E_2_ from the ovary and P_4_ from the corpus luteum regulate the expression of many endometrial genes during the cycle (4, 8), we hypothesized that E_2_ and P_4_ might affect the expression of caspases in the endometrium. Therefore, we obtained endometrial tissues from immature gilts, which had not been exposed to cyclical ovarian hormones, and treated them with increasing doses of E_2_ or P_4_. We found that the expression of *CASP7* mRNA was decreased by E_2_ (0 vs. 500 pg/ml, *P* < 0.05), but the expression of *CASP3, CASP6, CASP8, CASP9*, and *CASP10* mRNA was unaffected by E_2_ (Figure 5). The expression of *CASP7* (0 vs. 30 ng/ml, *P* < 0.05), *CASP8* (0 vs. 3 ng/ml and 0 vs. 30 ng/ml, *P* < 0.01), and *CASP10* (0 vs. 3 ng/ml, *P* < 0.01; 0 vs. 30 ng/ml, *P* < 0.05), but not *CASP3, CASP6*, and *CASP9*, was affected by P_4_ treatment (Figure 6).

### Effects of IL1B and Steroid Hormones on *CASP3* and the Effect of IFNG on *CASP7* Expression in Endometrial Tissues

**Figure 7:**
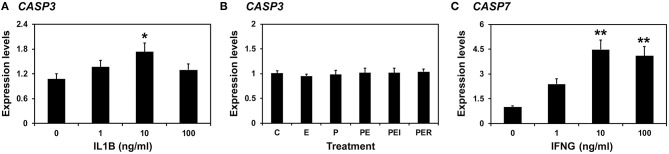
Effects of IL1B **(A)** and steroid hormones **(B)** on the expression of *CASP3* and the effect of IFNG on *CASP7* **(C)** in endometrial explant cultures. Endometrial explants from gilts on Day 12 of the estrous cycle were cultured **(A)** with increasing doses of IL1B (0, 1, 10, and 100 ng/ml) in the presence of with E_2_ (estradiol-17β; 10 ng/ml) and P_4_ (progesterone; 30 ng/ml), **(B)** with steroid hormones [control (C), E_2_ (E), P_4_ (P), E_2_ + P_4_ (PE), E_2_+P_4_+ICI (I, an estrogen receptor antagonist) (PEI), or E_2_+P_4_+RU (R; a progesterone receptor antagonist) (PER)], or **(C)** with increasing doses of IFNG (0, 1, 10, and 100 ng/ml) in the presence of with E_2_ (10 ng/ml) and P_4_ (30 ng/ml). The abundance of mRNA expression, determined by real-time RT-PCR analyses, was relative to that for *CASP3* and *CASP7* mRNA in the control group of endometrial explants after normalization to the transcript amounts of *RPL7, UBB*, and *TBP* mRNAs. Data are presented as means with standard error. These treatments were performed in triplicate using tissues obtained from each of three gilts. The asterisks denote statistically significant differences when values were compared with the control group: *P < 0.05; **P < 0.01.

Because the expression of *CASP3* and *CASP7* was highest on Days 12 and 15 of pregnancy, respectively, and porcine conceptuses secrete estrogen and IL1B2 into the uterine lumen on Day 12 and IFND and IFNG on Day 15 (4, 8), we assumed that the expression of *CASP3* on Day 12 could be affected by estrogen and IL1B and that of *CASP7* on Day 15 of pregnancy could be affected by IFNG. We treated endometrial explant tissues from Day 12 of the estrous cycle with increasing doses of IL1B and steroid hormones and found that IL1B induces the expression of *CASP3* (0 vs. 1 ng/ml, *P* < 0.05; Figure 7A), but steroid hormones and their receptor antagonists do not affect the expression of *CASP3* (Figure 7B). When increasing doses of IFNG were administered, the IFNG induced the expression of *CASP7* (0 vs. 10 pg/ml, 0 vs. 100 pg/ml; *P* < 0.01) (Figure 7C).

## Discussion

The significant findings of this study in pigs were: (1) caspases *CASP3, CASP6, CASP7, CASP8, CASP9*, and *CASP10* were expressed in the endometrium during the estrous cycle and pregnancy in a stage- and pregnancy status–specific manner; (2) conceptuses on Days 12 and 15 of pregnancy and chorioallantoic tissues from Day 30 of pregnancy to term expressed caspases, except *CASP9*, on Days 12 and 15 of pregnancy; (3) CASP3, cleaved-CASP3, and CASP7 proteins were localized to endometrial cells, with increased signal intensity in LE and GE cells during early pregnancy; (4) apoptotic cells in the endometrium were localized to some scattered stromal cells, with increased numbers on Day 15 of pregnancy; (5) E_2_ and P_4_ affected the expression of some caspases in endometrial tissues; and (6) IL1B and IFNG upregulated the expression of *CASP3* and *CASP7*, respectively, in endometrial explant tissues.

Caspases are essential mediators of apoptosis and play an important role in a variety of biological processes (9, 10, 17). Two groups of caspases, initiator caspases and executioner caspases, are activated during the pathway to apoptotic activation. Caspases are expressed in the endometrium during the reproductive cycle and pregnancy and that they mediate apoptotic cell death in various species (2, 30, 31). However, the expression of all initiator and executioner caspases in the endometrium throughout the estrous/menstrual cycle and at the maternal-conceptus interface during pregnancy has not been fully studied in any species. The results of this study indicate the variable expression of the initiator and executioner caspases in the endometrium during the estrous cycle and pregnancy and in conceptus/chorioallantoic tissues throughout pregnancy in pigs.

During the estrous cycle, the expression of caspases *CASP3, CASP6, CASP7, CASP8*, and *CASP10* changed with the stage of the cycle, with the highest levels in the proestrus phase for *CASP3, CASP6*, and *CASP7* and in the proestrus to metestrus phase for *CASP8* and *CASP10*. These data indicate that the expression of caspases is dynamically regulated in the endometrium during the estrous cycle and may be related to cyclic remodeling of this tissue in pigs. The incidence of apoptotic cell death in LE cells was previously shown by TUNEL assay to be highest in the estrus phase in pigs (14), suggesting that caspases expressed in the proestrus phase could cause apoptotic cell death in the endometrium in the estrus phase. In bovine endometrium, *CASP3* expression does not change during the estrous cycle, but active forms of CASP3 proteins increase at the follicular and early luteal phases compared with the mid- to late luteal phase (2). Furthermore, *CASP8* expression in the bovine endometrium increases toward the follicular phase from the luteal phase (31). Thus, it seems that the endometrial expression of some caspases increases in pigs and cows as the cycle moves toward the estrus phase.

The pattern of caspase expression in the endometrium during the estrous cycle led us to postulate that the expression of caspases and the activation of apoptotic signaling could be related to cyclical changes in the endometrium triggered by the actions of steroid hormones from the ovary. In this study, we found that P_4_ decreased the expression of *CASP8* and *CASP10* in endometrial explant tissues. Because the endometrial expression of *CASP8* and *CASP10* was low at the diestrus phase and high at the proestrus to metestrus phase of the estrous cycle, it is likely that P_4_ causes the decreased levels of *CASP8* and *CASP10* expression in the endometrium at the diestrus phase of the cycle in pigs. However, P_4_ increased the expression of *CASP7*, whereas E_2_ decreased the expression of *CASP7* in endometrial explant tissues, even though the endometrial expression of *CASP7* was high in the proestrus phase of the cycle, when plasma levels of P_4_ and E_2_ decrease and increase, respectively (8). These data indicate that the regulation of *CASP7* expression in the endometrium during the estrous cycle is much more complex than can be explained by the simple action of P_4_ and E_2_ and thus needs further analysis.

Although the levels of caspase expression during pregnancy have not been much studied in any species, it has been shown that the levels of active Casp3 protein in the rat endometrium are highest at mid-pregnancy (30). In this study, the endometrial expression of caspases *CASP3, CASP6, CASP7*, and *CASP10* during pregnancy changed, with the highest levels occurring during early pregnancy for *CASP3* and *CASP7* and during mid-pregnancy for *CASP6* and *CASP10*, suggesting that the expression of caspases is pregnancy stage–specific and varies with the type of caspase. In particular, we observed that the expression of *CASP3* and *CASP7* was highest on Days 12 and 15 of pregnancy, respectively, which is the period when conceptuses interact with the endometrium for implantation (4, 7, 8). Because the implanting porcine conceptus secretes estrogen and IL1B on Day 12 of pregnancy and type I and II IFNs, IFND and IFNG, around Day 15 of pregnancy (4, 8), we postulated that estrogen and/or IL1B might be responsible for inducing *CASP3* expression in the endometrium on Day 12 and that IFNG might be responsible for inducing *CASP7* expression on Day 15 of pregnancy. Indeed, our results performed using endometrial explant culture revealed increased expression of CASP3 in response to IL1B but not E_2_, whereas CASP7 expression was stimulated by IFNG. These data indicate that the expression of *CASP3* and *CASP7* in the endometrium during early pregnancy in pigs is induced by conceptus-derived IL1B and IFNG, respectively.

Because CASP3 and CASP7 are well-known executioner caspases during apoptosis (9, 17), we determined which cell type(s) expressed CASP3 and CASP7 proteins in the endometrium during early pregnancy and whether the cells expressing CASP3 and CASP7 were undergoing apoptotic cell death. Our results show that CASP3 and active CASP3 proteins were predominantly localized to LE and stromal cells on Days 12 and 15 of pregnancy and that the CASP7 protein was primarily localized to LE cells on Day 15 of pregnancy. Interestingly, however, results from the TUNEL assay and cleaved-PARP1 staining show that only stromal cells in the endometrium, not epithelial cells, were undergoing apoptosis during early pregnancy. These data suggest that CASP3 and CASP7 might not be involved in endometrial epithelial apoptosis during early pregnancy in pigs.

Executioner caspases CASP3, CASP6, and CASP7 play critical roles in both apoptotic cell death and cell differentiation in various cell types, such as keratinocytes, muscle cells, neurons, and stem cells (18). Also, the initiator caspase CASP8, which is expressed by cytotrophoblast cells, is involved in the differentiation of cytotrophoblast cells into the syncytiotrophoblast layer in human placental villi (32–34). Thus, the increased endometrial expression of CASP3 and CASP7 in response to conceptus-derived signals at the time of conceptus implantation could be expected to act on epithelial cell differentiation instead of activating apoptosis. Indeed, at the time of implantation LE cells of the porcine endometrium show various aspects of differentiated cellular characteristics: changed morphology (35–37), increased production of secretory proteins, including fibroblast growth factor 7 (38) and secreted phosphoprotein 1 (39), and increased expression of immunity-related molecules, including interferon α/β receptor 1 and 2 (40), interferon gamma receptor 1 and 2 (25), cysteine-X-cysteine motif chemokine ligand 12 (41), TNF superfamily 10 (28), and cytotoxic T-lymphocyte-associated protein 4 (Yoo and Ka, unpublished data). The expression of most of those molecules in endometrial epithelial cells is induced by conceptus signals, estrogen, IL1B, or IFNG, and those molecules play important roles in conceptus implantation. Thus, it is likely that CASP3 and CASP7 are also involved in activating the differentiation process of endometrial LE cells in response to conceptus-derived signals. However, the nature of the differentiated cellular characteristics mediated by CASP3 and CASP7 in endometrial LE cells still needs further study.

Our results also show that caspases were expressed in conceptus tissues during early pregnancy and in chorioallantoic tissues during mid- to late pregnancy. In particular, the levels of *CASP6, CASP7, CASP8, CASP9*, and *CASP10* expression in chorioallantoic tissues increased as the pregnancy came to term. However, as determined by TUNEL assay in this study, apoptotic cells were barely detectable in chorioallantoic tissues during pregnancy. It has been shown that CASP3 proteins levels in porcine placental tissue are higher on Day 30 than on Days 60, 80, and 90 of pregnancy, what coincides with the expression pattern of *CASP3* mRNA in this study (42). In ovine placentas, CASP3 and CASP9 proteins increase toward term, and the levels of active CASP3 and CASP9 are increased in placentas with intrauterine growth restriction pregnancy compared with normal pregnancy (43). In bovine placental tissues obtained at parturition, CASP3 and CASP8 mRNA and proteins are expressed (44), and CASP8 and CASP10 are expressed in human placental villi at term (33, 45). Thus, the expression of caspases in placental tissues is common among mammalian species and increases toward term. In addition, because accumulated evidence shows that apoptotic cell death increases in the placenta when pregnancy complications occur, such as intrauterine growth restriction, preeclampsia and preterm premature rupture of membranes in humans (46, 47), and in placental tissues derived from somatic cell nuclear transfer–cloned embryos in pigs (48), it is likely that caspases are important in regulating placental function and are activated in situations of inappropriate placental development during pregnancy.

## Conclusion

In conclusion, the results of this study in pigs show that caspases are expressed in the endometrium, with differential expression patterns throughout the estrous cycle and pregnancy, and in the conceptus and chorioallantoic tissues during pregnancy; CASP3 and CASP7 are localized primarily to endometrial epithelial cells during early pregnancy; the steroid hormones E_2_ and P_4_ regulate the expression of caspases in endometrial tissues; and IL1B and IFNG induce the expression of *CASP3* and *CASP7*, respectively, in endometrial tissues. These results suggest that caspases dynamically expressed in the endometrium and at the maternal-conceptus interface could play important roles in the establishment and maintenance of pregnancy in pigs by regulating apoptosis and epithelial differentiation.

## Data Availability Statement

The original contributions presented in the study are included in the article/Supplementary Material, further inquiries can be directed to the corresponding author/s.

## Ethics Statement

The animal study was reviewed and approved by Institutional Animal Care and Use Committee of Yonsei University.

## Author Contributions

WJ, MH, B-YJ, and HK: conceptualization, methodology. IY, JH, MK, SL, and YC: investigation. WJ, IY, and HK: data analysis, writing—original draft preparation. MH and B-YJ: writing—reviewing and editing. HK: supervision, funding acquisition. All authors contributed to the article and approved the submitted version.

## Conflict of Interest

The authors declare that the research was conducted in the absence of any commercial or financial relationships that could be construed as a potential conflict of interest.
