# Supplementary material for: Expression of Caspases in the Pig Endometrium Throughout the Estrous Cycle and at the Maternal-Conceptus Interface During Pregnancy and Regulation by Steroid Hormones and Cytokines
Source: Front Vet Sci. 2021 Feb 12;8:641916. doi: 10.3389/fvets.2021.641916 (PMC7907442; doi:10.3389/fvets.2021.641916)
Supplement: Supplementary file 1 [file Table_1.docx]

| **Supplementary Table. 1** Summary of primer sequences for RT-PCR and real-time RT-PCR and expected product sizes | | | | | | | | | |
| --- | --- | --- | --- | --- | --- | --- | --- | --- | --- |
| Primer | Sequence of forward (F) and  reverse (R) primers (5′ → 3′) | | Annealing temperature (℃) |  | Product size (bp) |  | No. of cycles |  | GenBank accession no. |
| *CASP3* |  | F: CTG GCA AAC CCA AAC TTT TC | 60 |  | 184 |  | 40 |  | AB029345.1 |
|  |  | R: GAT CCG TCC TTT GAA TTT CG |  |  |  |  |  |  |  |
| *CASP6* |  | F: TCA GAG CCT GGT TGG AAA AC | 60 |  | 243 |  | 40 |  | XM_005656547.3 |
|  |  | R: ATG TAC CAG GAG CCG TTC AC |  |  |  |  |  |  |  |
| *CASP7* |  | F: CCA GTC TTC TGT CCC TGC TC | 60 |  | 232 |  | 40 |  | XM_021073318.1 |
|  |  | R: TGC CCA CTT TCT CAA AAT CC |  |  |  |  |  |  |  |
| *CASP8* |  | F: ACA GTG ATG ACC TGG CTT CC | 60 |  | 211 |  | 40 |  | NM_001031779.2 |
|  |  | R: TCC TCC TCA TTG GTT TCC AG |  |  |  |  |  |  |  |
| *CASP9* |  | F: TCG ACT GTG AGA GGA TGC AG | 60 |  | 235 |  | 40 |  | XM_003127618.4 |
|  |  | R: ATT TTC TCC ACG GAC ACA GG |  |  |  |  |  |  |  |
| *CASP10* |  | F: GGG GGT TTT GTG TTG TTG TC | 60 |  | 160 |  | 40 |  | NM_001161640.1 |
|  |  | R: AGA ACC TGC ATG AGG TGG AC |  |  |  |  |  |  |  |
| *RPL7* |  | F: AAG CCA AGC ACT ATC ACA AGG AAT ACA | 60 |  | 172 |  | 40 |  | NM_001113217 |
|  |  | R: TGC AAC ACC TTT CTG ACC TTT GG |  |  |  |  |  |  |  |
| *UBB* |  | F: GCA TTG TTG GCG GTT TCG | 60 |  | 65 |  | 40 |  | NM_001105309.1 |
|  |  | R: AGA CGC TGT GAA GCC AAT CA |  |  |  |  |  |  |  |
| *TBP* |  | F: AAC AGT TCA GTA GTT ATG AGC CAG A | 60 |  | 153 |  | 40 |  | XM_013991786.1 |
|  |  | R: AGA TGT TCT CAA ACG CTT CG |  |  |  |  |  |  |  |
